# Supplementary material for: Patterns of Intron Gain and Loss in Fungi
Source: PLoS Biol. 2004 Nov 30;2(12):e422. doi: 10.1371/journal.pbio.0020422 (PMC532390; doi:10.1371/journal.pbio.0020422)
Supplement: Table S1 — Also available at http://genes.mit.edu/NielsenEtAl/. (4.3 MB ZIP). [file pbio.0020422.st001.zip › NielsenEtAl/html/1334.html]

AN7715.1.NCU06386.1.MG06137.1.FG06214.1


```
 CLUSTAL W (1.82) Multiple Sequence Alignments - Introns Inserted


Sequence 1: NCU06386.1	439 aa
Sequence 2: FG06214.1	414 aa
Sequence 3: MG06137.1	415 aa
Sequence 4: AN7715.1	405 aa
Alignment Length: 461 aa
Number Identitical Residues: 164 aa
Alignment Score (without introns) 8529


MG06137.1 	---MATHNGLY---ERFLWPAVDVVRYAPPHMILLGMLALLIPGFLA~LYFILHLVAPKP
NCU06386.1	MAPSSTISDIVGAPLQLVWGLYDVAAETSTPTLFLIAVSVAGLAFGL0IYIVLHIVAPKP
FG06214.1 	-MTVATPRELP----EFLRPLWAWIEATPVYVLVVLFIGLITLAVFE0LYVILHLVAPKP
AN7715.1  	-MPVTDWADIC-------VHCAELLTSVPLSIIIAAICATVAGVFVS0FYAFLTLVAPTP
          	  . :    :                 ..   :.    .     .   :* .* :***.*

MG06137.1 	RAPLPSEKTYLTSSPKDGRNTTAQLPCWYDRWLAEQRRQRQ-------------ANEDAP
NCU06386.1	RLPYPSEKTYITTNPDGTISKPRPLPCWYDRWHAESRLKEQSSSSTTVSYPTIPAGFPVP
FG06214.1 	RPVHPAEKQYITSHPTTGRTQPQPLPCWYDRWLAERQDSEQHIKPS--------EAYPTP
AN7715.1  	REPLPEETTYRTISKDGSITEPQSLPSWQR------------------------PGSNKA
          	*   * *. * *       . .  **.*                               .

MG06137.1 	DLGLIEPAEVAMSVVVPAYNEESRILPTLEEMVEYLDARFGR---DPPAKSTQTALVSPT
NCU06386.1	DFGTVEPAEVEISVVIPAYNEADRLIPTLEEMVAYLDEHFGRPAISGPGSRPTSKGTGTP
FG06214.1 	DAGNIEPAQVRLSVVFPAYNEEDRVIPTLVEAVAYLDEYFGR--TTAP---PTSSTSPSS
AN7715.1  	DPG---EAELFMSLVVPAYNEEDRLGGMLEEAVNYLERMYGTLASSRSEGKPLRQRK-TA
          	* *    *:: :*:*.***** .*:   * * * **:  :*  :   .   .      ..

MG06137.1 	TPKRLALKKHQAVPEPLT--GYEIIIVNDGSTDCTVQVALDFARDR--GLH---------
NCU06386.1	KPHRNVFKDASAHKRQHPPSGYEILLINDGSTDRTVDVALDFSRQH--NLH---------
FG06214.1 	KRHG-----RTAPKEELG--GYEILVVDDGSRDKTVDVVLQFAQEN--GLH---------
AN7715.1  	NGHANGIANSNTETTPHR-KGWEIIIVSDGSTDNTEEMAFSFARDHQLSLHPKGHAGPWT
          	. :     .  :       .*:**:::.*** * * ::.:.*:::.. .**... :.. :

MG06137.1 	-----------DIVRVVTLAKNRGKGGGVTHGFRHARGEYVVFADADGASKFSDLGKLVE
NCU06386.1	-----------DILRIVTLEKNRGKGGGVTHGLRHVRGEYAVFADADGASRFGDLGKLIE
FG06214.1 	-----------DILRVISLERNRGKGGATTHGFRHVRGEYILFADADGASRFCDAGKLIE
AN7715.1  	PEPREGVHIPPGTIRVVTLAKNRGKGGAVTHGMRHVRGQYVIFADADGATNFADLGKLVT
          	... ..   ... :*:::* :******..***:**.**:* :*******:.* * ***: 

MG06137.1 	GCEDVVDGSNRGVAIGSRGHLVGSEAVVK0RSALRNFLMKSFHFVLMILTPPATSRIRDT
NCU06386.1	GCEDVVDGSNRGVAIGSRAHLVGSEAVVK~RSAVRNFLMRSFHFVLMILTPPATSRIRDT
FG06214.1 	GCEEVVDGSYRGIAIGSRAHLVGSEAVVK0RSALRNFLMRSFHLVLMILTPPATSRIRDT
AN7715.1  	ACQEVEDSHGRGVAVGSRAHMVGSDAVVK0RSKLRNFLMHSFHLILWLLTPAKTAMIKDT
          	.*::* *.  **:*:***.*:***:**** ** :*****:***::* :***. *: *:**

MG06137.1 	QCGFKLFSRAALPHIIPYMHSEGWIFDIEMLMLAESAPATPVVAADGSVIGMSPGIKVVE
NCU06386.1	QCGFKLFSRAALPHIVPYMHTEGWIFDIEMLMLAESAPATPVLASDGSVIGTSYGIKVAE
FG06214.1 	QCGFKLFSRASLPHIIPYMHTEGWIFDIEMLMLAESAPATPVLGHDGSVIGTSPGIKVAE
AN7715.1  	QCGFKLFSRASLPAIVPYMHSEGWIFDVEMLMLAEFAR-----------------IPVAE
          	**********:** *:****:******:******* *                  * *.*

MG06137.1 	VPIDWHEVEGSKMNLIHDSIKMAIGLAVLRASWMMGVYRRRLT
NCU06386.1	VPIGWHEVGGSKMNLVKDSVKMAIGLAVLRASWMLGVYRRRLA
FG06214.1 	VPIEWHEVGGSKLNVIQDSIKMAIGLAVLRASWMFGVYRRRLT
AN7715.1  	VPVDWREVGGSKLNVIRDSVGMAWSLAVLRAAWLLGVYRRT--
          	**: *:** ***:*:::**: ** .******:*::*****
```
